# Therapeutic characteristics of alveolar-like macrophages in mouse models of hyperoxia and LPS-induced lung inflammation

**DOI:** 10.1152/ajplung.00270.2023

**Published:** 2024-06-18

**Authors:** Kymberly Litman, Sheena Bouch, Michael L. Litvack, Martin Post

**Affiliations:** ^1^Translational Medicine Programme, The Hospital for Sick Children, Toronto, Ontario, Canada; ^2^Department of Laboratory Medicine and Pathobiology, University of Toronto, Toronto, Ontario, Canada

**Keywords:** alveolar macrophage, ARDS, cellular therapy, hyperoxia, inflammation

## Abstract

Acute respiratory distress syndrome (ARDS) is a severe lung disease of high mortality (30–50%). Patients require lifesaving supplemental oxygen therapy; however, hyperoxia can induce pulmonary inflammation and cellular damage. Although alveolar macrophages (AMs) are essential for lung immune homeostasis, they become compromised during inflammatory lung injury. To combat this, stem cell-derived alveolar-like macrophages (ALMs) are a prospective therapeutic for lung diseases like ARDS. Using in vitro and in vivo approaches, we investigated the impact of hyperoxia on murine ALMs during acute inflammation. In vitro, ALMs retained their viability, growth, and antimicrobial abilities when cultured at 60% O_2_, whereas they die at 90% O_2_. In contrast, ALMs instilled in mouse lungs remained viable during exposure of mice to 90% O_2_. The ability of the delivered ALMs to phagocytose *Pseudomonas aeruginosa* was not impaired by exposure to 60 or 90% O_2_. Furthermore, ALMs remained immunologically stable in a murine model of LPS-induced lung inflammation when exposed to 60 and 90% O_2_ and effectively attenuated the accumulation of CD11b^+^ inflammatory cells in the airways. These results support the potential use of ALMs in patients with ARDS receiving supplemental oxygen therapy.

**NEW & NOTEWORTHY** The current findings support the prospective use of stem cell-derived alveolar-like macrophages (ALMs) as a therapeutic for inflammatory lung disease such as acute respiratory distress syndrome (ARDS) during supplemental oxygen therapy where lungs are exposed to high levels of oxygen. Alveolar-like macrophages directly delivered to mouse lungs were found to remain viable, immunologically stable, phagocytic toward live *Pseudomonas aeruginosa*, and effective in reducing CD11b^+^ inflammatory cell numbers in LPS-challenged lungs during moderate and extreme hyperoxic exposure.

## Graphical abstract

**Figure:**
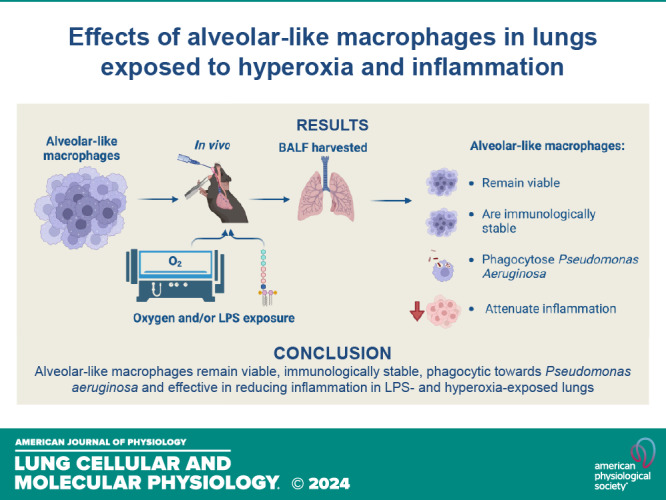

## INTRODUCTION

Acute lung injury (ALI) and its more severe form, acute respiratory distress syndrome (ARDS), continue to cause significant morbidity and mortality (1–3). A prospective cohort study conducted from 1999 to 2000 determined that ARDS develops in almost 200,000 patients yearly in the United States and is associated with an estimated 40% death rate (4). Although treatment strategies have improved over the years, there is still no effective cure for this life-threatening disease (5). ALI and ARDS are characterized by widespread pulmonary inflammation and increased pulmonary vascular permeability that ultimately leads to severe respiratory failure (5). Supplemental oxygen therapy remains the primary intervention for patients with severe ALI/ARDS; however, the extreme oxygen levels (0.5–1 $FI_{O_{2}}$) required for survival can be detrimental to pulmonary tissues and cells, risking additional pulmonary injury or a compromised immune response (6). In animal lung disease models, hyperoxia is often used to directly cause significant lung injury, and several studies have indicated that hyperoxia exacerbates preexisting lung injury (7–10). Inflammatory changes in the lung caused by hyperoxic exposure also frequently leads to an increased susceptibility to ventilator-associated pneumonia (VAP), most commonly caused by the major respiratory pathogen *Pseudomonas aeruginosa* (11). Development of secondary bacterial or viral infections can further complicate ALI/ARDS progression and prevent proper recovery. Bacterial VAP occurs in approximately one-third of patients with ARDS in intensive care units (ICUs), and the mortality rate of patients with ARDS with VAP is 57% (12, 13).

Alveolar macrophages (AMs) are lung-resident immune cells that play a critical role in maintaining tissue homeostasis and pulmonary innate immunity (14). During the progression of ARDS, AMs respond to signals in the lung microenvironment and modulate their functional phenotype to mediate the initiation and resolution of inflammation (15–20). Specifically, inflammatory activation of AMs drives the inflammatory phase of ARDS, whereas anti-inflammatory activation of AMs aids the resolution phase of ARDS (20, 21). In recent years, emerging studies have focused on how macrophages can be harnessed for therapeutic use (19, 22, 23). Their ability to eliminate pathogens and phenotypic versatility has made the concept of alveolar macrophage transplantation an attractive research focus for ARDS therapies (19, 23). However, acquiring healthy primary AMs poses a challenge due to difficulties in obtaining them in sufficient quantities and the necessity of invasive procedures (24). Prolonged exposure to hyperoxia has also been shown to exert harmful effects on lung structure and the viability, function, and inflammatory profile of AMs (25–29). This would represent a possible challenge if delivered to patients with ARDS who require supplemental oxygen therapy; thus, the effects of oxygen on AMs should be considered when investigating their use as a therapeutic strategy.

Recently, we generated mouse alveolar-like macrophages (ALMs) from pluripotent stem cells that resolve airway disease where the resident AM population was compromised (30). ALMs inactivate viruses and kill bacteria, resolve lung injury associated with viral and bacterial infections, and intratracheal administration of ALMs promotes repair in acute and chronic pulmonary injury (30–32). The therapeutic potential of ALMs has yet to be investigated in a high-oxygen environment that models patients with ARDS receiving supplemental oxygen therapy. In this study, we evaluated the viability, antimicrobial functions, and inflammatory/anti-inflammatory phenotype of ALMs after in vitro and in vivo exposure to moderate (60% O_2_) and extreme (90% O_2_) hyperoxia. We also characterized the phenotype of ALMs after hyperoxia exposure to LPS-induced lung inflammation in mice as the inflammatory state of alveolar macrophages can greatly influence ARDS severity. Our findings suggest that ALMs may have useful immunomodulatory functions that could have therapeutic applications to patients with ARDS receiving supportive oxygen therapy.

## MATERIALS AND METHODS

### Cell Culture and Maintenance

Mouse ALMs were differentiated as previously described by Litvack et al. (30) and cultured in serum-free medium supplemented with 5% (vol/vol) KnockOut Serum replacement (KOSR) containing 20 ng/mL granulocyte macrophage-colony stimulating factor (GM-CSF, R&D Systems) and 10 ng/mL macrophage-colony stimulating factor (M-CSF, R&D Systems). ALMs were passaged every 5 to 7 days at a 1:10 ratio using ReLeSR (Stem Cell Technologies) and maintained at 37°C in normoxia (21% O_2_) containing 5% CO_2_. Plates were moved to a separate hyperoxia incubator for exposure to 60% or 90% O_2_ with 5% CO_2_. Details of all cell culturing reagents can be found in Supplemental Table S1.

### Viability and Cell Growth Assay

We sought to evaluate the impact of both moderate (60% O_2_) and extreme (90% O_2_) hyperoxia on ALMs and resident AMs viability and proliferation in vitro. Resident AMs were collected by bronchoalveolar lavage (BAL) (as outlined in *Bronchoalveolar Lavage*) and isolated by allowing AMs to attach to normal cell culture plates overnight and removing nonadherent cells after 18 h (33). Next, ALMs and resident AMs were exposed to either normoxia (21% O_2_), 60% O_2_, or 90% O_2_ for 72 h. Attached and floating cells were collected, and cell growth was determined by use of standard hemocytometer. For in vitro viability experiments, cells were incubated for 5 min at 4°C with 3 μL 7AAD (7-Aminoactinomycin D) Viability Staining Solution (Invitrogen) per 100 μL of cell suspension. For in vivo viability experiments, cells collected by bronchoalveolar lavage (BAL) were stained with VivaFix 410/450 Viability dye (Bio-Rad) at a 1:1,500 dilution for 30 min at 4°C. Cells were washed and resuspended in sorting buffer containing HBSS (Life Technologies), 0.002% (wt/vol) HEPES (Sigma Aldrich), and 1% (vol/vol) FBS (Life Technologies) to determine cell viability by flow cytometry.

### Cell Stimulation and Macrophage Polarization

ALMs were polarized toward the pro-inflammatory phenotype in vitro by stimulation with 20 ng/mL mouse recombinant IFN-γ (Peprotech) and 10 ng/mL *Escherichia coli* LPS O26:B6 (Sigma Aldrich) for 24 h. Stimulation was performed without the presence of GM-CSF and M-CSF.

### Beads Uptake Assay

To determine whether ALMs can efficiently perform phagocytic functions after hyperoxia exposure, a phagocytic assay was performed using fluorescent latex beads after 72 h of 21% or 60% O_2_ exposure in vitro. One microliter of 1 μm blue-fluorescent beads (Invitrogen) was incubated in 1 mL of 4% (wt/vol) BSA (Gibco) for 75 min at 37°C on a Thermomixer R set to 900 rpm. BSA-coated beads were then added at 15 μL per 1 mL of culture medium to adherent ALMs that had been preexposed for 72 h to 21% or 60% O_2_. The 12-well plates were centrifuged for 1 min at 100 *g* and then incubated in 21% or 60% O_2_ at 37°C for 2 h. As a negative control, ALMs were incubated with beads at 4°C for 2 h to inhibit phagocytosis. Wells were washed with PBS before collection of cells for flow cytometry or immunostaining. Bead uptake by ALMs was determined as double-positive expression of blue fluorescence and murine macrophage marker F4/80 (APC) by flow cytometry. To visualize bead uptake by immunostaining, ALMs harvested using ReLeSR (StemCell) were resuspended in PBS for a final concentration of 1 × 10^5^ ALMs per 200 μL. A cytospot of 1 × 10^5^ ALMs was created using a cytospin centrifuge (Thermo Scientific Cytospin 4 Cytocentrifuge). ALMs were fixed using 4% (vol/vol) PFA for 20 min at room temperature (RT). ALMs were washed with PBS three times, and a blocking solution containing 10% (vol/vol) normal donkey serum and 1% (wt/vol) BSA in PBS was added for 60 min at RT. Primary antibody staining was performed overnight in a humidified staining tray at 4°C using rat anti-mouse F4/80 antibody (Abcam) at a dilution of 1:200. The ALMs were washed with PBS three times before incubation with goat anti-rat Alexa Fluor 647 secondary antibody (Invitrogen) at a dilution of 1:200 for 60 min at RT. After washing three times with PBS, microscope slides were mounted with Mowiol mounting medium (Sigma Aldrich) and imaged by Leica CTRMIC 6000 confocal microscope (Leica Microsystems). Images were analyzed with Volocity Software (Perkin Elmer Inc., v.6.3).

### Internalization of Fluorescent Bacteria

After 72 h of exposure to either 21% or 60% O_2_, ALMs were harvested using ReLeSR and were plated in 8-well chamber slides at 5 × 10^4^ ALMs per well and stained with FarRed Cell Trace (Invitrogen) for 20 min at RT. After washing with PBS twice, live green fluorescent protein (GFP)-expressing *P. aeruginosa* (PAO1pMF) (green) bacteria were added to the wells for a multiplicity of infection (MOI) of 10 bacteria per ALM (10:1) and the chamber slides were returned to either 21% or 60% O_2_ for 3 h. Wells were washed twice with PBS and fixed with 4% (vol/vol) PFA for 20 min. Wells were washed again with PBS, and nuclei were counterstained with DAPI for 20 min at RT. PBS was added to the wells, and images were taken with a Leica CTRMIC 6000 confocal microscope (Leica Microsystems). Images were analyzed with Volocity Software.

### Gentamicin Protection Assay

To assess ALMs’ ability to kill internalized bacteria, a gentamicin protection assay (GPA) was performed (32). We determined whether ALMs’ bactericidal efficiency would be retained after exposure to moderate hyperoxia, by directly comparing ALMs exposed to either 21% or 60% O_2_ for 72 h in antibiotic-free conditions. ALMs were harvested using ReLeSR and resuspended in MEM (Gibco) with 0.5% (vol/vol) KOSR for seeding in a 96-well plate with 1.5 × 10^5^ cells per well. *Pseudomonas aeruginosa* PAO1 were grown in 3 mL of LB broth (Invitrogen) in a round-bottle tube for 17 h on a bacterial shaker at 220 rpm at 37°C. The gentamicin protection assay was performed as described by Bouch et al. (32).

### Flow Cytometry

Cells were collected in sorting buffer, and to block the FcγII receptor, cells were incubated with purified anti-mouse CD16/32 (Biolegend) at a dilution of 1:50 for 5 min. To characterize cell surface markers on ALMs, fluorescently conjugated primary antibodies were added accordingly for 30 min at 4°C. To characterize intracellular markers, ALMs were fixed and permeabilized using the Intracellular Fixation and Permeabilization Buffer Set as described by the manufacturer (Invitrogen). Fluorescently conjugated primary antibodies for intracellular targets were added accordingly for 30 min at RT. Details of all antibodies used and concentrations are outlined in Supplemental Table S2. Unstained cells were used as a negative control. Cells were washed and resuspended in sorting buffer for analysis with Beckman Coulter Gallios 10/3 Flow Cytometer. All acquisitions were analyzed using Kaluza Analysis Software.

### In Vivo Model of Hyperoxia and/or LPS-Induced Lung Inflammation

All experiments were conducted under the approval of the Animal Care Committee of The Hospital for Sick Children (AUP No. 51434). Male C57BL/6 mice, 6–8 wk old, were supplied by Charles River Canada. To track ALMs in vivo, they were stained with FarRed Cell Trace for 30 min at RT. ALMs were washed with PBS and resuspended in PBS for a final concentration of 4 × 10^6^ ALMs/100 μL. Mice were weighed and anesthetized by intraperitoneal injection of 20 mg/kg ketamine (Vetoquinol) and 10 mg/kg xylazine (Rompun). Tracheal intubation was performed with a 24-gauge angiocath. Mice received a low-dose intratracheal instillation of 1 mg/kg *E. coli* LPS O26:B6 (Sigma Aldrich) to induce acute lung inflammation (Supplemental Fig. S1*A*). After 30 min, FarRed-ALMs were intratracheally instilled into the lungs in two boluses of 50 μL. Each instillation was administered 30 min apart. Control groups received intratracheal instillations of 50 μL PBS (vehicle only). Each concentration of oxygen exposure included four experimental groups: PBS + PBS, PBS + ALMs, LPS + PBS, and LPS + ALMs. Approximately 1 h after instillation, mice were exposed to 60 or 90% O_2_ for 24 or 72 h using BioSpherix OxyCycler A42OC chambers. Control groups were exposed to normoxia (21% O_2_). Oxygen exposure remained uninterrupted except for 3–5 min daily for animal health monitoring and to ensure that no oxygen toxicity was observed. All mice had access ad libitum to food and water and were monitored twice daily. After oxygen exposure, a BAL was performed and cells in the BAL fluid (BALF) were prepared accordingly for flow cytometry analysis and the supernatant was collected and stored at −80°C for subsequent IL-6 analysis.

### Internalization of *P. aeruginosa* by ALMs during Mouse Hyperoxia Exposure

Mice were anesthetized with the ketamine-xylazine mixture and received intratracheal instillation of FarRed-stained ALMs. Mice were then exposed to 21% O_2_, 60% O_2_, or 90% O_2_ for 72 h as previously described. After 72 h of oxygen exposure, mice were exposed for 4 min to 2% isoflurane. A 50 μL bolus of *P. aeruginosa* (ATCC, No. PAO1pMF230) containing a GFP plasmid No. Addgene ID: Plasmid No. 6246, PAO1pMF) at an MOI of 10 bacteria/1 ALM (10:1) in PBS was intratracheally instilled into the lungs (Supplemental Fig. S1*B*). The mice were immediately returned to their respective oxygen chambers for 3 h prior to euthanasia and BAL collection. BALF cells were prepared accordingly for flow cytometry analysis and cytospot immunostaining.

### Bronchoalveolar Lavage

Mice were euthanized with an overdose of euthanyl/pentobarbital sodium (Bimeda-MTC) by intraperitoneal injection. The diaphragm, sternum, and anterior ribs were dissected to expose the thoracic area and allow lung visualization. The neck tissue was dissected to expose the trachea and a small incision was created to allow passage of a 24-gauge angiocath into the trachea. Surgical suture was used to stabilize the angiocath and the lungs were lavaged with cold PBS using a 1-mL syringe. Lungs were lavaged three times with 1 mL aliquots of PBS drawn in and out twice, for a total of six washes per mouse. Approximately, 3 mL of lavage fluid was recovered per mouse. The collected BALF was centrifuged at 400 *g* for 20 min. The cell pellets were resuspended in sorting buffer for subsequent staining and flow cytometry analysis as described earlier.

### Uptake of *P. aeruginosa* by ALMs and Primary Alveolar Macrophages

Bronchoalveolar lavage of two healthy untreated mice was performed to collect primary alveolar macrophages. ALMs were separately collected from in vitro cell cultures kept at 21% O_2_. Both primary AMs and ALMs were resuspended in MEM (Gibco) with 0.5% (vol/vol) KOSR and seeded in a 96-well plate with 1.5 × 10^5^ cells per well. *Pseudomonas aeruginosa* (ATCC, No. PAO1pMF230) containing a GFP plasmid No. Addgene ID: Plasmid No. 6246, PAO1pMF) was added to the wells at an MOI of 10 bacteria/1 ALM (10:1) for 3 h of co-incubation. Cells were collected for flow cytometry; AMs were characterized by F4/80-positive staining, and bacterial uptake by ALMs or AMs was determined by their GFP-positive expression.

### In Vivo Characterization of ALMs and Primary BALF Cells

To identify ALMs in BALF, ALMs were stained with a CellTrace FarRed dye prior to intratracheal instillation as described earlier. Flow cytometry gating for FarRed^+^ cells was used to identify ALMs in vivo (Supplemental Fig. S2). We looked at changes in the endogenous inflammatory cell response by identifying inflammatory cell types in BALF including neutrophils and monocytes. Endogenous BALF cells were analyzed by gating for the FarRed^−^ population. Total inflammatory cell recruitment was determined by FarRed^−^/CD11b^+^ expression that was then further characterized as neutrophils (GR-1^+^/CCR2^−^) or monocytes (GR-1^+^/CCR2^+^).

### BALF Interleukin-6 Analysis

To determine the pro-inflammatory cytokine IL-6 content in the airways of our in vivo hyperoxia and/or LPS-exposed mice, BALF was collected 24 h after oxygen exposure and LPS and/or ALM administration. BALF supernatants were analyzed for IL-6 using a mouse IL-6 ELISA kit (Abcam, ab100712). In brief, supernatant samples were incubated in a 96-well plate coated with IL-6 antibodies overnight at 4°C. Following washing, biotinylated anti-mouse IL-6 antibodies were added. Next, after washing away unbound antibody, HRP-conjugated streptavidin was added, followed by a stop solution, and the amount of IL-6 was determined by reading the absorbance at 450 nm. Data were plotted in relation to the standard curve. All samples and standards were run in duplicate, and IL-6 expression was normalized to the total protein content of each sample, as determined by a Bradford assay (Bio-Rad, as per the manufacturer’s instructions).

### Statistical Analysis

All data are expressed as means ± standard error of mean (SE). Comparisons between two groups were made using a *t* test with Welch’s correction. Comparisons between more than two groups were made using one-way, two-way, three-way, or repeated-measures ANOVA as appropriate followed by Tukey’s multiple comparisons test. GraphPad Prism V. 9 was used to perform the appropriate statistical analyses.

## RESULTS

### ALMs Are Viable and Proliferate under Moderate, but Not Extreme, Hyperoxia In Vitro

**Figure 1.**
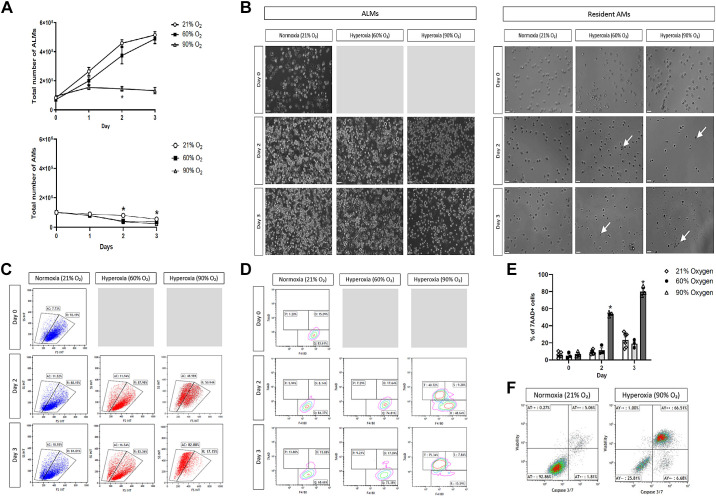
Effects of hyperoxia on alveolar-like macrophages (ALMs) growth and death in vitro. *A*: growth of ALMs (*top*) and resident alveolar macrophages (AMs) (*bottom*) during 3 days of exposure to normoxia (21% O_2_), moderate (60% O_2_), or extreme (90% O_2_) hyperoxia. Total number of ALMs and resident AMs were counted on each day of exposure (*n* = 3 biological replicates, means ± SE, sexes were equally represented). *P < 0.05, one-way repeated-measures ANOVA. *B*: representative brightfield images of ALMs and resident AMs during 3 days of exposure to normoxia or hyperoxia. Arrows indicate cell blebbing and death. Images of ALMs and resident AMs were taken at ×50 and ×40 magnification, respectively. Scale bar is 60 μm. *C*: representative flow cytometry FSC/SSC plots of ALMs during 3 days of exposure to normoxia or hyperoxia (*n* ≥ 3 biological replicates). *D*: representative flow cytometry contour plots of F4/80- and 7AAD-stained ALMs over 3 days of exposure to normoxia or hyperoxia. *E*: percentage cell death after exposure of ALMs to 21%, 60%, or 90% O_2_ for 3 days measured by 7AAD flow cytometry (*n* = 3 biological replicates, means ± SE). *P < 0.05, one-way repeated-measures ANOVA. *F*: representative flow cytometry plots depicting apoptotic (caspase 3/7-positive) ALMs after 48 h of 21% or 90% O_2_ exposure. −−, viable cells, +−, early apoptotic cells, ++, late apoptotic cells (*n* = 3 biological replicates).

To evaluate the effect of hyperoxia on viability and cell growth of ALMs and resident AMs, cells were collected daily during 72 h of exposure to 21%, 60%, or 90% O_2_ in vitro. Although ALMs did not increase in cell numbers after 1 day in extreme (90% O_2_) hyperoxia, ALMs did grow under normoxia (21% O_2_) and moderate (60% O_2_) hyperoxia. There was no significant difference in ALM proliferation over 3 days between the moderate hyperoxia and normoxia groups (Fig. 1*A*, *top*). Conversely, after 1 day, the number of resident AMs decreased in all three groups (21%, 60%, and 90% O_2_), and on *day 2* and *3*, the number of AMs was significantly decreased in the 60% and 90% O_2_ groups compared with the normoxia group (Fig. 1*A*, *bottom*). ALMs exposed to moderate hyperoxia had comparable morphology to ALMs exposed to normoxic conditions (Fig. 1*B*, *left*), whereas the AMs exposed to 60% and 90% oxygen showed considerable amounts of cell blebbing and death (Fig. 1*B*, *right*). Flow cytometry characterization of the resident AMs could not be performed satisfactorily due to the loss of cell integrity and viability (data not shown). Forward and side scatter gating during flow cytometry analysis confirmed a marked decrease in cell size of ALMs exposed to 90% but not 60% O_2_ (Fig. 1*C*), suggesting cell death. ALMs were analyzed by flow cytometry for viability 7AAD and macrophage F4/80 markers. Contour plots revealed separate populations of viable (F4/80^hi^, 7AAD^−^), early apoptotic (F4/80^hi^/7AAD^lo^), and dead (F4/80^lo^, 7AAD^hi^) ALMs (Fig. 1*D*). After 60% O_2_ exposure for 3 days, ALMs were indistinguishable from ALMs cultured in 21% O_2_ (*n* = 6, *P* = 0.5746). Conversely, 90% O_2_ exposure led to significantly increased ALM death [53.79 ± 1.51% (*n* = 3), *P* < 0.0001 by *day 2* and ∼80.06 ± 3.39% (*n* = 3), *P* < 0.0001 by *day 3*] (Fig. 1*E*). After 48 h of 90% O_2_ exposure, ALMs were also double positive for Caspase 3/7 and viability stain VivaFix 410/450 [58.11 ± 5.34% (*n* = 3)], indicating rapid activation of apoptosis (Fig. 1*F*). For the remainder of the in vitro experiments, 60% O_2_ was used to assess ALMs behavior under hyperoxic conditions because ALMs were not viable when exposed to 90% O_2_.

### ALMs Phagocytose Beads and Bacteria under Moderate Hyperoxia In Vitro

**Figure 2.**
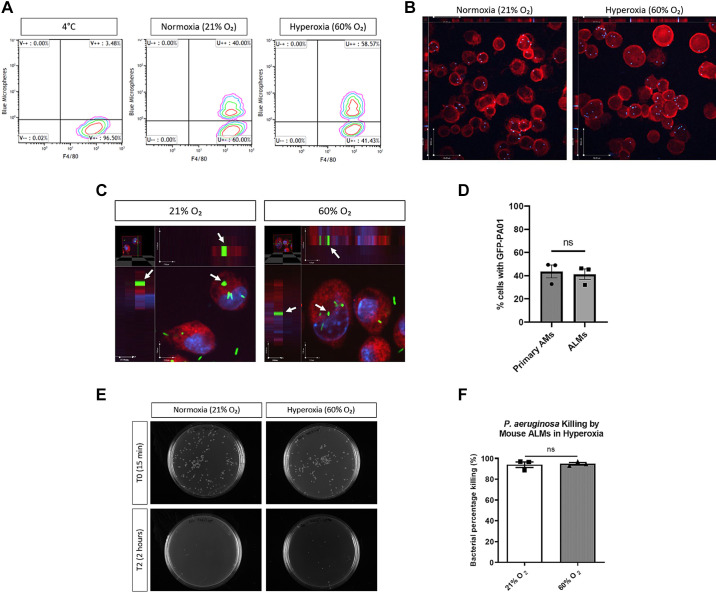
Moderate hyperoxia does not alter alveolar-like macrophage (ALM)s’ phagocytotic and bactericidal characteristics in vitro. *A*: percentage of F4/80^+^ ALMs, exposed for 72 h to 21% and 60% O_2_, phagocytosing blue-fluorescent beads as assessed by flow cytometry contour plots (*n* = 3 biological replicates). *B*: respective confocal images display the uptake of blue-fluorescent beads by ALMs identified by primary F4/80 and secondary Alexa Fluor 647 antibodies. Images were taken at ×40 magnification. Scale bars are 50 μm. *C*: confocal microscopy of FarRed-stained ALMs internalizing live green fluorescent protein (GFP)-expressing *Pseudomonas aeruginosa* under 21% and 60% O_2_ conditions. Cells were counterstained with DAPI and images were taken at ×40 magnification with multilayered *Z* stack. White arrows represent internalized bacteria. Scale bars are 7 μm (*y*-axis) and 7 μm (*x*-axis). *D*: the percentage of F4/80-stained primary alveolar macrophages (AMs) and FarRed-stained ALMs containing GFP-expressing *P. aeruginosa* following 3 h of co-incubation [multiplicity of infection (MOI) = 10:1] under normoxia in vitro. Primary AMs were obtained by bronchiolar lavage of healthy untreated mouse lungs. *E*: bactericidal activity is shown by reduced bacterial CFU on agar plates from T0 (15 min) to T2 (2 h) of incubation with gentamicin. *F*: comparison of bacterial killing capacity of ALMs toward *P. aeruginosa* after 72 h exposure to 21% and 60% O_2_ (*n* = 3 biological replicates carried out in triplicate, means ± SE). ns, nonsignificant; unpaired *t* test.

We first evaluated the uptake of blue-fluorescent latex beads by ALMs after 72 h of exposure to 21% and 60% O_2_. The population of ALMs that phagocytosed beads was distinguished by flow cytometry as cells double positive for F4/80 and blue fluorescence, whereas F4/80^+^ cells without blue fluorescence represented ALMs that had not taken up any beads. ALMs and beads co-incubated at 4°C did not display any phagocytosis of fluorescent beads (Fig. 2*A*). The percentage of hyperoxia-exposed ALMs phagocytosing fluorescent beads after co-incubation at 37°C was comparable with that of ALMs kept in 21% O_2_ (Fig. 2*A*). Internalization of blue-fluorescent beads by ALMs cultured at 21% and 60% O_2_ was confirmed by confocal microscopy (Fig. 2*B*).

In addition to phagocytosis of fluorescent latex beads, we evaluated ALMs’ ability to internalize bacteria. ALMs exposed for 72 h to 21% or 60% O_2_ were co-incubated with live GFP-fluorescent *P. aeruginosa* PAO1 bacteria for 3 h and imaged by confocal microscopy. Internalization of the bacteria by hyperoxia-exposed ALMs was comparable with ALMs kept in 21% O_2_ (Fig. 2*C*). In vitro, the phagocytotic activity of ALMs for live *P. aeruginosa* bacteria was similar to primary AMs freshly isolated from healthy unexposed mice (Fig. 2*D*).

### ALMs Kill Bacteria under Moderate Hyperoxia In Vitro

Previously, we reported that ALMs are efficacious at killing various laboratory and clinical strains of bacteria using a standard GPA (32). Here, *P. aeruginosa* PAO1 bacteria were used to determine ALMs’ bactericidal efficiency after hyperoxia exposure. ALMs demonstrated a high capacity of killing (93.99 ± 2.74%, *n* = 3) toward *P. aeruginosa* under normoxic conditions (Fig. 2*E*). ALMs exposed to 60% O_2_ retained that high killing capacity against *P. aeruginosa* (94.88 ± 1.18%, *n* = 3, *P* = 0.7872) (Fig. 2*F*).

### Phenotype of ALMs under Moderate Hyperoxia In Vitro

**Figure 3.**
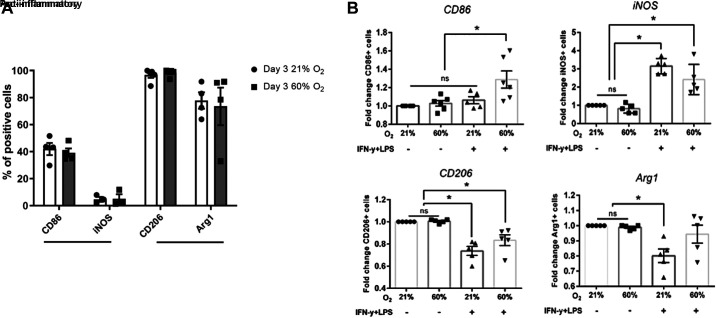
Moderate 60% hyperoxia does not alter alveolar-like macrophage (ALM)’s inflammatory/anti-inflammatory status in vitro. *A*: expression of inflammatory/anti-inflammatory markers on ALMs assessed by flow cytometry after 72 h exposure to 21% and 60% O_2_ (*n* ≥ 3 biological replicates, means ± SE). *B*: ALMs polarization toward inflammatory cells when stimulated with LPS + IFN-γ for 24 h under 21% or 60% O_2_ conditions. Graphs depict fold change of percentage positive cells for pro-inflammatory [CD86, inducible nitric oxide synthase (iNOS)] and anti-inflammatory (CD206, Arg1) markers relative to unstimulated ALMs kept at 21% O_2_ (*n* ≥ 5 biological replicates, means ± SE). *P < 0.05, two-way ANOVA.

We have previously characterized ALMs in vitro by inflammatory markers inducible nitric oxide synthase (iNOS) and CD86 and anti-inflammatory markers Arg1 and CD206 (32). Here, we determined whether moderate hyperoxia alters the phenotype of the ALMs in vitro. Flow cytometry was used to investigate the expression of the markers on ALMs after culturing for 72 h at 21% O_2_ or 60% O_2_. Under normoxic conditions, a high percentage of ALMs express Arg1 (77.70 ± 6.05%, *n* = 4) and low percentage express iNOS (4.72 ± 1.81%, *n* = 3) (Fig. 3*A*). The percentage of CD206^+^ cells was also high (98.79 ± 0.50%, *n* = 5), whereas an intermediate percentage of cells were CD86^+^ (42.04 ± 4.52%, *n* = 4) (Fig. 3*A*). Overall, ALMs in normoxic conditions appear to have an anti-inflammatory phenotype in vitro, consistent with our previous findings (32). After exposure to 60% O_2_ for 72 h, no significant changes in ALMs’ marker expression were observed (Fig. 3*A*). To observe whether an inflammatory environment combined with hyperoxia would alter ALMs’ phenotype, ALMs were stimulated with LPS + IFN-γ for 24 h under either 21% or 60% O_2_ conditions. Under normoxic conditions, LPS + IFN-γ significantly increased the percentage of iNOS^+^ cells [3.14 ± 0.19 fold change vs. unstimulated ALMs (*n* = 5), *P* < 0.0001 (Fig. 3*B*)]. In addition, ALMs expressing anti-inflammatory markers Arg1 and CD206 were reduced [0.801 ± 0.045 fold change (*n* = 5), *P* = 0.0079 and 0.73 ± 0.04 fold change (*n* = 5), *P* = 0.0001, respectively (Fig. 3*B*)]. Similar changes in number of CD206^+^ and iNOS^+^ ALMs after LPS + IFN-γ challenge were observed in ALMs cultured in 60% O_2_. LPS + IFN-γ exposure did not significantly alter the percentage of Arg1^+^ ALMs in hyperoxia-exposed ALMs although a trend toward reduced expression was noted. Percentage of CD86^+^ ALMs was not changed by LPS + IFN-γ stimulation in 21% O_2_ but was significantly increased in 60% O_2_ [1.28 ± 0.09 fold change (*n* = 5), *P* = 0.0057 (Fig. 3*B*)].

### ALMs Remain Viable in Mice Exposed to Extreme Hyperoxia Alone but Not in Combination with LPS

**Figure 4.**
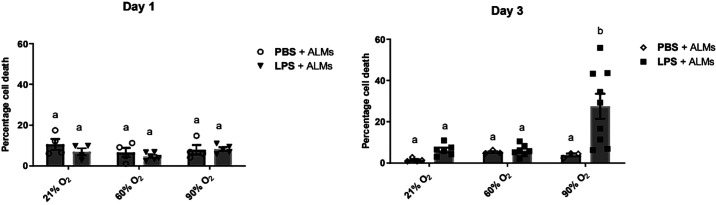
Viability of alveolar-like macrophages (ALMs) during LPS-induced inflammation and hyperoxia exposure in vivo. Viability of FarRed-stained ALMs instilled in vehicle control (PBS) or LPS-treated lungs of male mice that were subsequently exposed to hyperoxia for 1 and 3 days was assessed by flow cytometry using VivaFix 410/450 viability dye (*n* ≥ 3 mice/group, means ± SE). Bars showing the same letter are not significantly different (*P* < 0.05, two-way ANOVA).

Studies have previously determined that prolonged exposure to hyperoxia can compromise the viability of endogenous AMs (28). Therefore, we evaluated whether hyperoxia alone, LPS alone, or a combination of both would be deleterious to the viability of ALMs in vivo. Flow cytometry analysis of BALF cells revealed that instilled ALMs remained viable in mice exposed to either 21%, 60%, or 90% O_2_, and upon LPS challenge 1 day after instillation (Fig. 4). Contrary to our in vitro findings, ALMs also remained viable in the airways of mice exposed to extreme hyperoxia of 90% O_2_ for 3 days. ALMs were additionally viable in LPS-challenged mice exposed to 21% and 60% O_2_ for 3 days. However, cell death of ALMs in LPS-challenged mice was significantly increased [3.84 ± 0.74% (*n* ≥ 3) to 27.52 ± 6.02% (*n* ≥ 3), *P* = 0.0413] after exposure to 90% O_2_ for 3 days (Fig. 4). These data suggest that ALMs remain viable during in vivo exposure to 90% O_2_ alone, but that cell death of ALMs is increased in LPS-challenged mice exposed for longer periods to 90%, but not 21% or 60% O_2_.

### ALMs Internalize *P. Aeruginosa* in Mice Exposed to Moderate and Extreme Hyperoxia

We also determined whether ALMs remain proficient at bacterial uptake during in vivo exposure to hyperoxia. ALMs were stained with a FarRed fluorescent dye to distinguish them from endogenous mouse BALF cells. Primary AMs in BALF were identified by gating on FarRed^−^ F4/80^+^ cells. Among FarRed^+^ cells, GFP^+^ fluorescence was assessed to compare the percentage of ALMs that had internalized GFP-expressing *P. aeruginosa* in mice exposed to different oxygen concentrations. We found that the percentages of ALMs that internalized live *P. aeruginosa* were similar between mice exposed to 21%, 60%, and 90% O_2_ (Table 1). Cytospot analyses of mouse BALF by confocal microscopy confirmed internalization of GFP^+^ *P. aeruginosa* by FarRed^+^ ALMs under all oxygen conditions (Supplemental Fig. S3). Thus, there was no significant change in the efficiency of ALMs bacterial uptake in situ during exposure of mice to moderate or extreme hyperoxia.

**Table 1.** Phagocytosis of ALMs in situ not altered by moderate and extreme hyperoxia

O_2_ Concentration	% FarRed^+^ ALMs with GFP	% FarRed^−^ F4/80^+^ AMs with GFP
21% O_2_	29.55 ± 28.14	12.18 ± 12.05
60% O_2_	35.30 ± 27.55	14.12 ± 14.39
90% O_2_	32.96 ± 10.21	24.70 ± 6.04

In vivo uptake of GFP-*Pseudomonas aeruginosa* by FarRed^+^ alveolar-like macrophages (ALMs) and FarRed^−^ primary alveolar macrophages (AMs) in bronchoalveolar lavage fluid (BALF) of male mice exposed to moderate (60% O_2_) and extreme (90% O_2_) hyperoxia. Values expressed as means ± SE, *n* ≥ 4 mice.

### Inflammatory Cell Response following Intratracheal LPS and Hyperoxia Exposure

**Figure 5.**
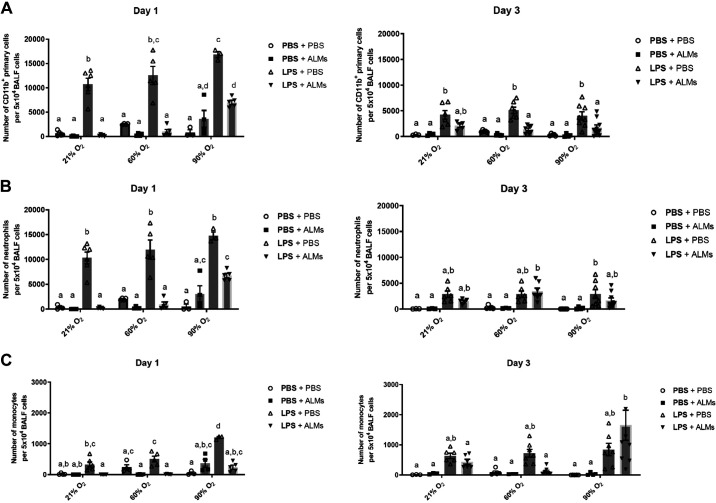
Inflammatory cell recruitment to the airways after LPS-induced acute lung injury and hyperoxia exposure. *A*: total inflammatory cell recruitment determined by flow cytometry of CD11b^+^ endogenous cells in bronchoalveolar lavage fluid (BALF) of male mice exposed to LPS (or PBS vehicle) with and without hyperoxia in the presence or absence of alveolar-like macrophages (ALMs). *B*: inflammatory neutrophil recruitment determined as GR-1^+^, CCR2^−^ cells among CD11b^+^ endogenous cells in mouse BALF. *C*: inflammatory monocyte recruitment determined as GR-1^+^, CCR2^+^ cells among CD11b^+^ endogenous cells in mouse BALF. Data are shown as means ± SE, *n* ≥ 3 mice/group. Bars showing the same letter are not significantly different (*P* < 0.05, two-way ANOVA).

In mice that received PBS or ALMs alone, CD11b^+^ cell recruitment to the BALF during either 21%, 60%, or 90% O_2_ exposure was minimal or absent. A significant increase in total CD11b^+^ cells was observed following intratracheal delivery of LPS alone (Fig. 5*A*). In the LPS + PBS 21% O_2_ group, recruitment of inflammatory CD11b^+^ cells peaked at *day 1* (10,788 ± 149 CD11b^+^ cells per 5 × 10^4^ BALF cells, *n* ≥ 3) and declined thereafter (Fig. 5*A*). The recruitment of CD11b^+^ cells at *day 1* was similarly increased in the LPS + PBS 60% O_2_ group. However, at *day 1* after LPS instillation, the LPS + PBS 90% O_2_ group displayed significantly greater numbers of CD11b^+^ cells (16,817 ± 598 per 5 × 10^4^ BALF cells, *n* ≥ 3, *P* = 0.0042) in the BALF compared with the LPS + PBS 21% O_2_ group (Fig. 5*A*). Mice in the LPS + ALM treated groups had significantly reduced numbers of CD11b^+^ cells in BALF (220.20 ± 3.35 CD11b^+^ cells per 5 × 10^4^ BALF cells, *n* = 5, *P* < 0.0001) compared with the LPS alone 21% O_2_ group; levels returned to that seen in PBS + ALM 21% O_2_ mice (Fig. 5*A*). This reduction in recruitment of CD11b^+^ cells to the BALF in LPS + ALM mice was also seen with additional exposure of mice to 60% or 90% O_2_, although this reduction occurred to a lesser extent at 90% O_2_, specifically after one day of exposure (Fig. 5*A*). The LPS-recruited CD11b^+^ cells to the BALF were primarily GR-1^+^/CCR2^−^ neutrophils (76.84 ± 3.98%) and the number of neutrophils was significantly reduced in LPS + ALMs treated mice compared with LPS + PBS treated mice independent of hyperoxia exposure (Fig. 5*B*). Although GR-1^+^/CCR2^+^ monocytes were only a small proportion (4.49 ± 0.64%) of the recruited CD11b^+^ cells, we observed a small, yet significant, influx of monocytes in LPS-treated mice exposed to either normoxia or hyperoxia_,_ which was significantly reduced in mice that received ALMs (Fig. 5*C*). Of note, the number of GR-1^+^/CCR2^+^ monocytes in BALF increased unexpectedly in the LPS + ALM-treated group after 3 days of 90% O_2_ exposure compared with PBS + ALMs 90% O_2_ group although numbers are relatively minor. Altogether, these results indicate that instillation of ALMs to mice with LPS-induced pulmonary inflammation reduced their total number of inflammatory cells in BALF during exposure to either 21%, 60%, or 90% O_2_.

### Phenotype of ALMs in a Mouse Model of LPS-Induced Lung Inflammation Exposed to Hyperoxia

**Figure 6.**
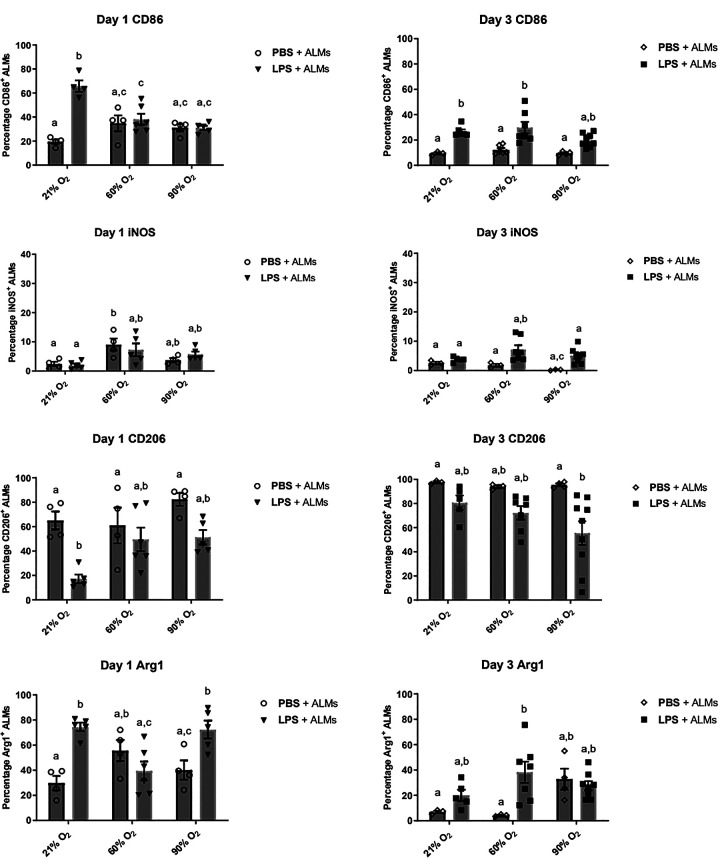
Inflammatory/anti-inflammatory marker expression of alveolar-like macrophages (ALMs) in situ after LPS-induced inflammation and hyperoxia exposure in vivo. Expression of inflammatory [CD86 and inducible nitric oxide synthase (iNOS)] and anti-inflammatory (CD206 and Arg1) markers was assessed by flow cytometry of FarRed-labeled ALMs recovered from bronchoalveolar lavage fluid (BALF) of male mice prechallenged with LPS (or PBS vehicle) and exposed to hyperoxia. (*n* ≥ 3 biological replicates, means ± SE). Data are shown as means ± SE, *n* ≥ 3 mice/group. Bars showing the same letter are not significantly different (*P* < 0.05, two-way ANOVA).

To determine changes in phenotype of ALMs in vivo, inflammatory and anti-inflammatory markers were assessed on FarRed^+^ ALMs collected from BALF of mice at 1 and 3 days after LPS delivery and exposure to hyperoxia. We first focused on the phenotypic response of ALMs at 1 day after instillation, because at this timepoint we observed the greatest cellular inflammatory response in the airways to LPS that was attenuated by ALMs (Fig. 5). We observed a significantly reduced percentage of CD206^+^ ALMs isolated from LPS-treated mice that were kept in 21% O_2_, whereas ALMs expressing Arg1 and CD86 were significantly increased (Fig. 6). When mice were additionally exposed to 60% O_2_, we no longer observed any significant changes in ALMs expressing inflammatory (CD86, iNOS) and anti-inflammatory (CD206, Arg1) markers in response to LPS (Fig. 6). During 90% O_2_ exposure, LPS-treated mice showed a similar increased percentage of Arg1^+^ ALMs as observed during normoxic exposure. However, we did not see any alteration in CD206^+^ and CD86^+^ ALMs after LPS delivery during 90% O_2_ exposure, nor did we observe any significant changes in iNOS^+^ ALMs in response to LPS with and without hyperoxia. We then looked at ALMs’ phenotypic response to LPS in vivo after 3 days of 21%, 60%, and 90% O_2_ exposure. Independent of hyperoxia, a minor, yet significant, increase in the percentage [9.42 ± 0.86% (*n* ≥ 3) vs. 26.66 ± 1.68% (*n* = 6), *P* < 0.0001] of CD86 expressing ALMs was observed (Fig. 6). Nonsignificant trends of increased iNOS and decreased CD206 expressing ALMs were noted, whereas Arg1 expressing ALMs was significantly increased in LPS-treated mice kept at 60%, but not 90% O_2_. Overall, the data show that there are minor changes in the phenotype of instilled ALMs after direct challenge of mouse lungs with LPS. The ALM phenotype was not further altered upon exposure of the mice to 60% and 90% O_2_.

### IL-6 Content in the BALF of Mice following Intratracheal LPS and Hyperoxia Exposure

First, IL-6 was determined in the BALF of mice exposed for 24 h to LPS and/or hyperoxia (both 60% and 90% O_2_). We observed a trend, but not significant, for BALF IL-6 to be increased in the LPS+PBS 21% O_2_ group compared with the PBS+PBS 21% O_2_ control group. However, IL-6 in the BALF was significantly increased in the LPS+PBS 60% O_2_ and LPS+PBS 90% O_2_ compared with the 21% O_2_ groups, demonstrating that hyperoxia exposure exacerbates LPS-induced IL-6 production (Supplemental Fig. S4*A*). Following confirmation of elevated BALF IL-6 in response to LPS and hyperoxia, we focused on investigating whether ALMs can attenuate IL-6 in BALF of lungs exposed to LPS and 90% O_2_ (Supplemental Fig. S4*B*). ALM administration (LPS+ALM 90% O_2_) attenuated BALF IL-6 so that it was no longer significantly different to the PBS+ALM 90% O_2_ group; however IL-6 in the BALF did remain significantly increased compared with the PBS+PBS 90% O_2_ group.

## DISCUSSION

Numerous studies have shown that hyperoxia causes cell death, functional impairment, and increased inflammatory activity of immune cells including AMs (25–28). AMs are major players in mediating the resolution of ALI/ARDS, and our previous studies have shown that stem cell-derived ALMs are a plausible therapeutic intervention during pulmonary disease (10, 30–32, 34). As an essential step toward their prospective immunotherapeutic use in patients receiving supplemental oxygen therapy, we investigated the effects of high oxygen exposures on the viability and biological function of the ALMs. Here, we demonstrate that ALMs instilled in mouse lungs remain viable and functional under moderate (60% O_2_) and extreme (90% O_2_) hyperoxia as well as under LPS challenge of the lungs followed by moderate and extreme hyperoxia exposure.

Our initial experiments showed that ALMs remained viable during exposure to moderate hyperoxia in vitro. ALMs proliferated and remained viable during 3 days of culture at 60% O_2_, but not at 90% O_2_. Increased Caspase 3/7 signaling suggests that ALMs endured apoptotic cell death upon 90% O_2_ exposure. In contrast to ALMs, resident AMs did not proliferate or remain viable when exposed to either 60% or 90% O_2_ in culture. These findings align with other reports, showing that extreme hyperoxia reduces AM viability and proliferation rates (35, 36). Despite reduced viability at 90% O_2_ in vitro, ALMs remained viable in vivo when mice were subjected to 3 days of 90% O_2_ exposure. We speculate that the robust endogenous lung antioxidant system in vivo neutralized hyperoxia-induced reactive oxygen species (ROS), thereby preventing the ALM cell death observed in vitro (25, 37, 38), though our study did not specifically focus on this.

Bacterial colonization can exacerbate pulmonary inflammation during ARDS, prolonging the recovery processes or possibly leading to death. Therefore, we aimed to address whether moderate (60% O_2_) hyperoxia would influence the bactericidal abilities of ALMs. Morrow et al. (25) have reported that primary AMs exhibit remarkably reduced bactericidal capabilities due to impaired phagocytic function in mice exposed to 65% O_2_ for 3 days. In contrast, we found no significant difference in phagocytosis of beads or killing of PAO1 between ALMs cultured at 60% O_2_ versus 21% O_2_ in vitro. Previously, we have shown that ALMs in 21% O_2_ also effectively kill clinical strains of *P. aeruginosa* and promote airway recovery in an in vivo model of *P. aeruginosa*-induced lung injury (32). Here, we found that, in 21% O_2_, ALMs are just as efficient as primary AMs at internalizing live *P. aeruginosa* and that phagocytosis of live *P. aeruginosa* by ALMs in situ was not affected by hyperoxia exposure. Our observations that ALMs retain their phagocytic abilities during in vitro and in vivo hyperoxia suggest that ALMs have greater bactericidal abilities in hyperoxia than primary AMs (25). Collectivity, these findings highlight their therapeutic value for patients receiving supplemental oxygen therapy.

In this study, we characterized the inflammatory phenotype of ALMs during hyperoxia in vitro. Several groups have reported that hyperoxia causes an aberrant phenotype of murine AMs and other lung macrophage populations (10, 27, 28, 39). However, these in vitro and in vivo experiments used extreme (≥85%) oxygen concentrations. We found that the ALM phenotype was not altered by 3 days of exposure to 60% O_2_. We also exposed ALMs to 60% O_2_ during LPS+IFN-γ stimulation to observe the influence of moderate hyperoxia during inflammatory activation. Contrary to our expectation that hyperoxia would promote pro-inflammatory activation in vitro, we did not observe any further increase in cells expressing inflammatory markers (iNOS) or decrease in cells expressing anti-inflammatory markers (CD206, Arg1). Future studies would be worthwhile to assess pro-inflammatory cytokines like IL-6 or IL-1β. Overall, the inflammatory state of ALMs was not perpetuated during additional exposure to 60% O_2_ in vitro.

Considering patients with ARDS are given high concentrations of oxygen for extended time periods, we aimed to characterize the changes in viability and phenotype of ALMs in an in vivo model of pulmonary inflammation with subsequent exposure to hyperoxia. To assess whether moderate or extreme levels of hyperoxia alter the inflammatory response of ALMs delivered to the lungs of LPS-challenged mice, we administered a low dose of LPS directly to the lungs, followed by 3 days of exposure of the mice to either 60% or 90% O_2_. In LPS-challenged mice, ALMs remained viable at *day 1* in mice exposed to either 21%, 60%, or 90% O_2_. At *day 3*, cell death of ALMs was significantly increased only in mice exposed to LPS and 90% O_2_ but not 60% O_2_. This may indicate that prolonged exposure to extreme but not moderate hyperoxia increases the vulnerability of ALMs to cell death in an inflammatory lung environment. However, in clinical practice, such extreme oxygen levels are frequently minimized to moderate concentrations once it is safe to do so to limit oxygen toxicity (25).

Our results additionally show that the peak of inflammatory cell recruitment to the airways occurred 1 day after LPS delivery, which was greatly reduced by 3 days. At *day 1*, 90% O_2_ resulted in a heightened accumulation of CD11b^+^ cells in the airways of LPS-challenged mice and increased IL-6 secretion, suggesting that extreme hyperoxia exacerbates airway inflammatory cell recruitment. This result correlates with previous studies showing that hyperoxia promoted CD11b^+^ cell recruitment to alveoli (28). We did not see any additive effects of moderate or extreme hyperoxia on inflammatory cell recruitment at *day 3*, likely due to the inflammatory resolution that has started. Increased IL-6 secretion and the prevalence of high numbers of inflammatory cells such as neutrophils in the BALF at *day 1* could also explain why we observed some significant changes in the phenotype of ALMs in response to LPS at *day 1*. This includes decreased CD206^+^ ALMs and increased Arg1^+^ ALMs, which we did not observe during normoxia by *day 3*. Neutrophils are known to produce pro-inflammatory cytokines such as IL-6, IL-8, IFN-γ, and TNF-α that perpetuate the inflammatory environment during lung injury (40). This pro-inflammatory cytokine signaling can reduce the expression of CD206, a hallmark anti-inflammatory marker on macrophages, a potential reason why a decrease in CD206 expressing ALMs was observed. Moreover, IFN-γ production can also downregulate CD206 expression (41). Conversely, the production of high levels of IL-6 by neutrophils has been implicated in enhancing the polarization to anti-inflammatory macrophages (42). This may explain the observed increase in Arg1^+^ ALMs. Surprisingly, both 60% and 90% O_2_ exposure did not augment the polarization of ALMs in response to LPS even at *day 1* in vivo. Instead, CD86^+^ ALMs remained unchanged during LPS stimulation in 90% O_2_, and the changes in CD206^+^ and Arg1^+^ ALMs by LPS in 21% O_2_ remained stable during 90% O_2_. Furthermore, there was no significant change in ALMs expressing the pro-inflammatory marker iNOS in response to LPS and hyperoxia. Although we expected LPS-induced inflammation to push ALMs toward a pro-inflammatory state and for this to be exacerbated by hyperoxia, our results indicate only moderate changes, characteristic of simultaneous expression of both inflammatory and anti-inflammatory markers that resolves back to baseline when inflammatory resolution occurs. Importantly, the phenotypic changes remained stable under the influences of moderate or extreme hyperoxia. This stable, hybrid plasticity could be valuable therapeutically since overt changes toward either a highly inflammatory or anti-inflammatory state during ALI/ARDS progression can contribute to a more pathological condition. For example, a dominant anti-inflammatory state of macrophages can promote fibrosis during chronic obstructive pulmonary disease (COPD) or idiopathic pulmonary fibrosis (IPF) (43). In contrast, a dominant inflammatory macrophage state has been shown to promote inflammatory damage during ALI/ARDS (43).

Finally, we observed significantly reduced numbers of CD11b^+^ cells in BALF of mice that received ALMs one day following LPS delivery under all oxygen conditions. Under normoxia and moderate hyperoxia conditions, instillation of ALMs in the lungs of mice was able to resolve the level of LPS-recruited CD11b^+^ cells in the airways back to normoxic baseline levels. In mice exposed to 90% O_2_, instillation of ALMs still produced a similar, albeit less drastic result yet was still able to attenuate IL-6 secretion in the lungs. It is unclear whether ALMs are clearing recruited CD11b^+^ cells through phagocytosis or inducing changes in paracrine signaling factors involved in neutrophil or monocyte recruitment to the airways. Subsequent studies should aim to clarify this by examining the phagocytosis of recruited cells by ALMs and determining the levels of key recruitment chemokines (e.g., MIP-2, KC, or MCP-1) in BALF. The recruitment of inflammatory CD11b^+^ cells has been implicated in exacerbating inflammation in the context of ARDS (44). The CD11b^+^ population is primarily compromised of activated neutrophils and produce large amounts of ROS and pro-inflammatory mediators that lead to pulmonary damage. Thus, the ability of ALMs to reduce the overall number of activated inflammatory cells in the airways is a promising characteristic for reducing overall inflammatory injury.

The lack of effective drug treatments for ALI/ARDS has led to a flood of investigations into cell-based therapies over the past decade (45–47). Although mesenchymal stem cells (MSCs) are the primary cell type that has been clinically studied for ARDS, they remain controversial because of the need for an abundant source, their undifferentiated state, and their short half-life (46, 48). In all these aspects, ALMs would be advantageous due to their *1*) high scalability in vitro, *2*) differentiated phenotype resembling lung resident AMs, and *3*) ability to persist in mouse airways (30). A recent study also showed that MSCs worsened ARDS in lung environments with high IL-6 and low antioxidant capacity, proving that MSCs can be detrimental to lung injury depending on the microenvironment during administration (49). Furthermore, ALMs contrast with MSCs due to their functional ability to phagocytose and kill pathogens that would be beneficial considering patients with ARDS are prone to secondary infections (31, 32). To the best of our knowledge, study by Liu et al. (23) is the only other study that has examined the delivery of macrophages for ALI/ARDS. They delivered IL-4 secreting RAW264.7 macrophages to an ALI mouse model (23). We believe ALMs would be a better candidate for macrophage therapy in airway disease over RAW264.7 cells, since RAW264.7 cells are a monocyte macrophage cell line while ALMs are specialized to reside in the alveolar niche. Importantly, these in vivo studies were not conducted with hyperoxia exposure in mind, so the influence of high oxygen in these cellular therapy models for ALI/ARDS is still unknown. Overall, our findings establish that ALMs show promising potential as a cellular therapy for patients with ALI/ARDS receiving oxygen therapy due to their functional and immunological stability during acute inflammation and hyperoxia exposure.

Despite the current findings, there are some limitations to our study. Although we looked at conventional markers to assess the phenotypic state of ALMs and to identify airway inflammatory cells, we did not identify secreted cytokines or signaling pathways involved in oxidative stress or inflammation. In addition, this study did not investigate whether hyperoxia affects ALMs’ ability to resolve endpoints of acute lung injury, such as increased wet/dry ratios, increased protein permeability, or histological evidence of damage. Future studies should investigate larger cohorts of mice and observe additional endpoints of lung injury in the presence of hyperoxia and delivery of ALMs during inflammatory lung disease.

## DATA AVAILABILITY

The data that support the findings of this study are available from the corresponding author upon reasonable request.

## SUPPLEMENTAL MATERIAL

10.6084/m9.figshare.25134656 Supplemental Figs. S1–S4 and Supplemental Tables S1 and S2: https://doi.org/10.6084/m9.figshare.25134656.

## GRANTS

This work was supported by Canadian Institutes of Health Research Grant FDN-143309 (to M.P.) and National Sanitarium Association IPR (to M.P.).

## DISCLOSURES

No conflicts of interest, financial or otherwise, are declared by the authors.

## AUTHOR CONTRIBUTIONS

K.L., S.B., M.L.L., and M.P. conceived and designed research; K.L. and S.B. performed experiments; K.L. and S.B. analyzed data; K.L., S.B., M.L.L., and M.P. interpreted results of experiments; K.L. and S.B. prepared figures; K.L. drafted manuscript; K.L., S.B., M.L.L., and M.P. edited and revised manuscript; K.L., S.B., and M.P. approved final version of manuscript.
